# Supplementary material for: IGF-1 rs6218 polymorphisms modulate the susceptibility to age-related cataract
Source: PeerJ. 2024 Apr 9;12:e17220. doi: 10.7717/peerj.17220 (PMC11011587; doi:10.7717/peerj.17220)
Supplement: Supplemental Information 3 [file peerj-12-17220-s003.doc]

**Gender**

1=male

2=femalle

Type

1=C: cortical cataract

2=N: nuclear cataract

3=PSC: posterior sub capsular cataract

4=M: mixed cataract
