# Supplementary material for: IGF-1 rs6218 polymorphisms modulate the susceptibility to age-related cataract
Source: PeerJ. 2024 Apr 9;12:e17220. doi: 10.7717/peerj.17220 (PMC11011587; doi:10.7717/peerj.17220)
Supplement: Supplemental Information 4 [file peerj-12-17220-s004.doc]

STROBE Statement—Checklist of items that should be included in reports of ***case-control studies***

|  | Item No | Recommendation |
| --- | --- | --- |
| **Title and abstract** | 1 | (*a*) The present investigation is a case-control study. |
|  |  | (*b*) Apoptosis of lens epithelial cells (LECs) is closely related to Age-related cataracts (ARC) formation. Insulin-like growth factor1 (IGF1) contributes to cell apoptosis regulation. Single nucleotide polymorphisms (SNPs) contribute to ARC development |
| Introduction | | |
| Background/rationale | 2 | Age-related cataract (ARC) is a multifactorial disorder standing as the primary contributor to blindness globally, especially in middle-income and low-income countries. Although surgery can restore vision in most patients, the procedure itself has some risks and complications. The risk of ARC was found to be affected by several genetic and environmental factors, including oxidative damage and metabolic disorders, among others.  Genetic variations, especially single nucleotide polymorphisms (SNPs), participate significantly in ARC development; several genes and SNPs were revealed to be associated with ARCs |
| Objectives | 3 | The definite pathogeny of ARC remains incompletely understood.  Whether SNPs of *IGF1* were related to ARC is still not clear. |
| Methods | | |
| Study design | 4 | to examine the correlation between polymorphisms in the *IGF1* and the susceptibility to ARC |
| Setting | 5 | 20 ARNC patients and 20 age-, sex- and ethnically matched controls From January 2022 to December 2022 |
| Participants | 6 | (a) The criteria of our epidemiological investigation for the ARC group was LOCSIII>C2; >N2; >P2, while the control group was LOCSIII≤C1; ≤N1; ≤P1.  We excluded the patients (both cases and controls) who had lens trauma, diabetes, uveitis glaucoma and high myopia (>6D) |
|  |  | (b) age-, sex- and ethnically-matched controls |
| Variables | 7 |  |
| Data sources/ measurement | 8* |  |
| Bias | 9 |  |
| Study size | 10 | In theory, the larger the number of participants in the epidemiological survey, the better. The number of participants we are willing to participate in is among our Jiangsu eye disease population |
| Quantitative variables | 11 |  |
| Statistical methods | 12 | (*a*) The t-test was utilized to conduct statistical comparisons between the average values of the two groups. The χ2 test was employed for evaluating the relationship between the allele frequencies of ARC patients and normal controls, different ARC subtypes, odds ratios (OR), and 95% confidence intervals (CI), and also for testing Hardy-Weinberg Equilibriums (HWE) of genotype distributions |
|  |  | (*b*) |
|  |  | (*c*) |
|  |  | (*d*) |
|  |  | (*e*) |
| Results | | |
| Participants | 13* | (a) Epidemic survey population：716 ARC patients (C = 377, N = 223, P = 48, M = 68) and 685 controls  inpatients in our hospital：20 ARNC patients and 20 age-, sex- and ethnically matched controls |
|  |  | (b) Subjective unwillingness of patients |
|  |  | (c) |
| Descriptive data | 14* | (a) |
|  |  | (b) |
| Outcome data | 15* |  |
| Main results | 16 | (*a*) |
|  |  | (*b*) |
|  |  | (*c*) |

| Other analyses | 17 |  |
| --- | --- | --- |
| Discussion | | |
| Key results | 18 | We found that the G allele frequency in the *IGF1*-rs6218 was higher in the ARCs than in the controls. Furthermore, it was observed that the rs6218 GG genotype exhibited a positive correlation to elevated levels of *IGF1* mRNA in LECs. The *IGF1* mRNA in the LECs and the apoptosis of LECs in N type of ARCs (ARNC) was higher than the controls |
| Limitations | 19 | there are many reasons for the apoptosis of LECs, which may not be solely the result of *IGF1* upregulation. |
| Interpretation | 20 | Further experiments are needed to detect apoptosis by enhancing or knocking out the *IGF1* gene in vitro experiments |
| Generalisability | 21 | The susceptibility to ARC is related to *IGF1*-rs6218 polymorphism, and this polymorphism is associated with *IGF1* expression at the mRNA level. Moreover, apoptosis in LECs of ARNC was found to be increased. |
| Other information | | |
| Funding | 22 | the Scientific research project of Changzhou Medical Center of Nanjing Medical University (CZKYCMCB202225); Science and Technology Project of Changzhou (CJ20220097;CE2022507); Project of Changzhou Health Commission(QN202129;ZD202120); Young Talent Development Plan of Changzhou Health Commission(CZQM2022017; CZQM2020091); Jiangsu Province Traditional Chinese Medicine Technology Development Project(MS2022078) |

*Give information separately for cases and controls.

**Note:** An Explanation and Elaboration article discusses each checklist item and gives methodological background and published examples of transparent reporting. The STROBE checklist is best used in conjunction with this article (freely available on the Web sites of PLoS Medicine at http://www.plosmedicine.org/, Annals of Internal Medicine at http://www.annals.org/, and Epidemiology at http://www.epidem.com/). Information on the STROBE Initiative is available at http://www.strobe-statement.org.
